# Sustainable Electrochemical Activation of Self-Generated Persulfate for the Degradation of Endocrine Disruptors: Kinetics, Performances, and Mechanisms

**DOI:** 10.3390/toxics12020156

**Published:** 2024-02-17

**Authors:** Xiaofeng Tang, Zhiquan Jin, Rui Zou, Yi Zhu, Xia Yao, Mengxuan Li, Shuang Song, Shuangliu Liu, Tao Zeng

**Affiliations:** 1 Key Laboratory of Microbial Technology for Industrial Pollution Control of Zhejiang Province, College of Environment, Zhejiang University of Technology, Hangzhou 310032, China; txf872350286@163.com (X.T.); 2111927020@zjut.edu.cn (Z.J.); 2112127061@zjut.edu.cn (R.Z.); 211122270037@zjut.edu.cn (Y.Z.); 17363909209@163.com (X.Y.); lscs652559316@163.com (M.L.); ss@zjut.edu.cn (S.S.); 2 Chinese Academy of Environmental Planning, Beijing 100012, China; 3 Shaoxing Research Institute, Zhejiang University of Technology, Shaoxing 312000, China

**Keywords:** self-circulation, persulfate, BDD anode, activation of S_2_O_8_^2−^, electrochemical remediation

## Abstract

This study presents an electrolysis system utilizing a novel self-circulation process of sulfate (SO_4_^2−^) and persulfate (S_2_O_8_^2−^) ions based on a boron-doped diamond (BDD) anode and an activated carbon fiber (ACF) cathode, which is designed to enable electrochemical remediation of environmental contaminants with reduced use of chemical reagents and minimized residues. The production of S_2_O_8_^2−^ and hydrogen peroxide (H_2_O_2_) on the BDD anode and ACF cathode, respectively, is identified as the source of active radicals for the contaminant degradation. The initiator, sulfate, is identified by comparing the degradation efficiency in NaSO_4_ and NaNO_3_ electrolytes. Quenching experiments and electron paramagnetic resonance (EPR) spectroscopy confirmed that the SO_4_^−^· and ·OH generated on the ACF cathode are the main reactive radicals. A comparison of the degradation efficiency and the generated S_2_O_8_^2−^/H_2_O_2_ of the divided/undivided electrolysis system is used to demonstrate the superiority of the synergistic effect between the BDD anode and ACF cathode. This work provides evidence of the effectiveness of the philosophy of “catalysis in lieu of supplementary chemical agents” and sheds light on the mechanism of the generation and transmission of reactive species in the BDD and ACF electrolysis system, thereby offering new perspectives for the design and optimization of electrolysis systems.

## 1. Introduction

Advanced oxidation processes (AOPs) have emerged as a promising technique for the efficient removal of endocrine disruptors from wastewater, owing to their high oxidation efficacy and the absence of secondary pollutants [1,2]. For the diverse types of AOPs, electrochemical degradation [3], Fenton and Fenton-like reactions [4], photo-Fenton [5], photocatalysis [6], and ozonation [7] have been extensively studied. Among these, the electrochemical degradation process has been found to possess distinctive advantages in addressing complex water matrices [8], such as municipal wastewater [9], landfill leachate [10], and reverse osmosis concentrate [11], making it a highly desirable option for the treatment of endocrine disruptors in wastewater.

The electrochemical degradation process involves the use of a range of anodes, among which the boron-doped diamond (BDD) electrode has garnered significant attention attributed to its high overpotential for oxygen evolution, robustness, and chemical stability [12], which set it apart from conventional electrodes such as PbO_2_, Pt, SnO_2_, and Ti [13]. Additionally, the BDD electrode also possesses a wide potential window that facilitates the production of active species, including adsorbed hydroxyl radicals (·OH_ads_) and persulfate (S_2_O_8_^2−^) [14,15]. The latter is typically generated through the electrolysis of sulfate (SO_4_^2−^) [16] and is generally considered a non-radical process owing to the negligible impact of radical scavengers on contaminant removal rates [17]. However, the kinetics of anodic oxidation are relatively slow, and the process requires substantial energy consumption, given that the reaction primarily involves physisorbed radicals (·OH_ads_, S_2_O_8_^2−^) interacting with pollutants [18]. Coupling a cathode with the BDD anode to generate radicals with enhanced redox potentials and reduced energy consumption would be a viable method to overcome the aforementioned limitations. The activated carbon–persulfate or activated carbon fiber (ACF)–persulfate process represents a promising technology for water treatment, owing to its high adsorption capacity, metal-free nature, and large surface area [19]. Several studies have demonstrated the superiority of this process in activating S_2_O_8_^2−^ to produce sulfate radicals (SO_4_^−^·) [20,21,22], which are potent oxidants with a redox potential E_0_ (SO_4_^−^·/SO_4_^2−^) of 2.5–3.1 V [23]. However, as-prepared electrolysis systems typically require additional dosages of persulfates, and some electrolysis systems comprised of BDD and ACF electrodes have neglected the synergistic effect between the two components [24,25,26]. Moreover, the underlying mechanism of reactive species generation and transmission in the BDD and ACF electrolysis system is poorly understood.

The objective of this study is to establish a self-circulation system of sulfate and persulfate ions in the BDD and ACF electrolysis system to circumvent the addition of supplementary chemical agents and elucidate the involvement of electrogenerated sulfate radical species. The model pollutant bisphenol A (BPA) was employed to evaluate the performance of the BDD and ACF electrolysis system using the inert Pt electrode as a contrast. The amount of generated S_2_O_8_^2−^ and hydrogen peroxide (H_2_O_2_) is quantitatively estimated to demonstrate the enhanced accumulation of S_2_O_8_^2−^ on the BDD anode and its activation by ACF. The experiments were carried out in sulfate anolyte and compared to inert nitrate with the same pH and conductivity to confirm the participation of sulfate in the formation of S_2_O_8_^2−^ species. By estimating the degradation efficiency of BPA and the accumulation of S_2_O_8_^2−^ and H_2_O_2_ of the divided and undivided cell, we confirm that the activation of S_2_O_8_^2−^ on the ACF cathode exhibited superiority compared to the non-radical oxidation on the anode. The degradation efficiency of BPA and the accumulation of S_2_O_8_^2−^ and H_2_O_2_ were estimated in both the divided and undivided cells to confirm the superiority of the activation of S_2_O_8_^2−^ on the ACF cathode over the non-radical oxidation on the anode. These observations provide insight into the self-circulation process between sulfate and persulfate ions and suggest new strategies for designing and optimizing electrolysis systems, as the process is completely free from the addition of chemical reagents.

## 2. Materials and Methods

### 2.1. Materials and Reagents

A Nafion-117 cation exchange membrane for separating catholyte and anolyte was manufactured by Du Pont de Nemours & Co., Ltd. (Wilmington, DE, USA). A potentiostat providing direct current (DC) power was purchased from Shanghai Chenhua Science Technology Corp., Ltd., Shanghai, China. Unless otherwise specified, all chemical reagents were acquired from commercial suppliers and utilized without further purification. Sodium persulfate (PS, ≥99.5%), carbamazepine, norfloxacin, atrazine, phonel, methanol (99%), bisphenol A (BPA), sodium hydroxide (NaOH), atrazine, sulfuric acid (H_2_SO_4_), sodium sulfate (Na_2_SO_4_, ≥99.0%), Diammonium 2,2′-azino-bis (3-ethylbenzothiazoline-6-sulfonate) (ABTS), potassium titanium (IV) oxalate, tert-butanol (TBA, 99%), and boron-doped diamond electrode (BDD) electrodes (800 ppm boron, bipolar, 1 mm coating thickness) were obtained from J&K Chemical (Beijing, China). Activated carbon fiber (ACF) electrodes and platinum-plated titanium electrodes (25 mm × 50 mm × 1 mm) were obtained from Shanxi Kaida Chemical Ltd., Lvliang, China. DC power supply was obtained from Zhejiang Sako Electrical Group Co., Ltd., Leqing, China.

### 2.2. Experimental Procedures

In the undivided cell, the experimental device is composed of a 500 mL glass beaker and two electrodes. To enhance the conductivity of the solution, a supporting electrolyte of 50 mM Na_2_SO_4_ was added to the 250 mL solution. The initial pH of the solution was adjusted to predetermined values ranging from 2 to 6 with the aid of either H_2_SO_4_ or NaOH solution (0.1 M). Bisphenol A, carbamazepine, norfloxacin, atrazine, and phenol were selected as the pollutants to evaluate the efficiency of electrochemical activation, which were introduced into the solution at a concentration of 0.044 mM. Prior to the commencement of degradation, the mixture solution was stirred for 30 min with a magnetic rod at 300 rpm to achieve homogeneous distribution and obtain an adsorption–desorption equilibrium. The distance of the anode and the cathode was 2 cm, and the substrate material of the electrode was ACF felt (SA1000–1500), provided by Nantong Shuang Activated Carbon Filter Material Co., Ltd., China. Prior to use, the ACF was thoroughly washed with pure water and ethanol and cut to dimensions of 25 mm × 50 mm × 1 mm (0.20 ± 0.01 g). The total immersion area of the electrode in the electrolytic cell was 10 cm^2^ (25 mm × 40 mm). In the divided cell, the anode chamber and cathode chamber was divided by the cation exchange membrane (CEM) with volume of 250 mL; the other settings of the reactor are consistent with those of the single-chamber reactor. All experiments were conducted under a direct current (DC) power (0–20 V) at a determined current density, except for those experiments focused on the effect of current density (0–25 mA·cm^−2^). At predetermined intervals, 1 mL of the solution was collected and filtered through a filter membrane with a pore size of 0.45 μm to remove residuals and immediately mixed with 500 μL of methanol. To determine the main free radical species formed in the system, ABTS and potassium titanium (IV) oxalate were employed as chromogenic agents to detect the yield of persulfate and hydrogen peroxide without adding pollutants. Free radical quenching experiments were conducted by adding excessive methanol and TBA (500 mM). All experiments were conducted three times, and the typical values with standard errors (error bars) were presented. The temperature of all experiments was maintained at 298 K.

### 2.3. Analytical Methods

BPA quantification was performed via high-performance liquid chromatography (HPLC) using a Symmetry C18 column (150 mm × 4.6 mm × 5 mm, Agilent, Santa Clara, CA, USA) and a PDA detector (Waters 2998, Milford, MA, USA). The mobile phase consisted of methanol and ultrapure water in a 30:70 ratio, with a flow rate of 1 mL·min^−1^, and detection was carried out at a monitoring wavelength of 225 nm.

The concentration of residual S_2_O_8_^2−^ in the solution was determined using the ABTS colorimetric method. Specifically, a 5.0 mL aliquot of the solution was filtered through a 0.45 µm pore membrane, followed by the addition of 5 mL of Milli-Q water, 0.4 mL of ABTS (10 mM), and 0.2 mL of Co^2+^ (10 mM). The mixture was allowed to react for 10 min, yielding the green-colored ABTS radical cation, which was analyzed by UV/Vis spectrophotometry (UV-T6, Beijing Puxi Co., Ltd., Beijing, China) at the maximum absorbance wavelength (λ_max_) of 735 nm.

The concentration of H_2_O_2_ was determined by the potassium titanium (IV) oxalate method, where 1.5 mL of the solution was drawn from the reactor using a syringe with a 0.45 µm filter at a given time interval and then mixed with 1 mL of a 0.02 M potassium titanium (IV) oxalate solution. The solution then changed from transparent to yellow due to the formation of Ti peroxides which was analyzed by UV/Vis spectrophotometry (UV-T6, Beijing Puxi Co., Ltd., China) at the maximum absorbance wavelength (λ_max_) of 400 nm.

The electronic paramagnetic resonance (EPR) was used to detect the active species. Exactly 100 mM 5,5-Dimethyl-1-pyrroline-n-oxide (DMPO) was added to the sample as capture agents to capture active species. The concentration of H_2_O_2_ was determined by the method of potassium titanium (IV) oxalate with a UV–vis spectrophotometer (SHIMADZU UV 2600) at 400 nm. The energy consumption (Ec, kWh·m^−3^) was assessed using the electrical efficiency per log order reduction (EE/O, Equation (1)) [27,28,29,30]:

(1) $$EE/O=\frac{I\times T\times U}{V\times \log\frac{C_{0}}{C}}$$

where I represents the applied current (A), T represents the reaction time (h), U is the average cell voltage (V), V is the volume (L) of the reaction system, C_0_ is the initial concentration of contaminants (mg·L^−1^), and C is the concentration of contaminants at reaction time t (mg·L^−1^).

## 3. Result and Discussion

### 3.1. Comparative Degradation of BPA in Different Electrode Systems

**Figure 1:**
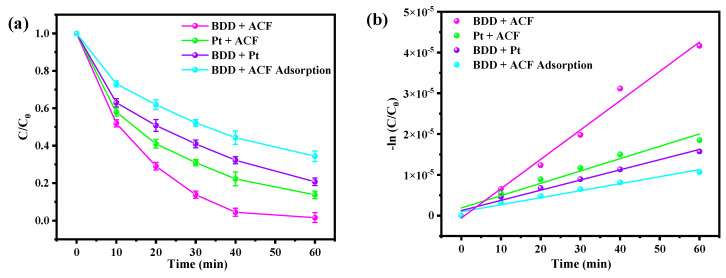
The degradation efficiency of BPA in the electrolysis system with different electrodes (**a**) and their corresponding reaction kinetics (**b**). (Initial pH = 2; temperature = 298 K; stirring speed = 800 rpm; initial BPA concentration = 0.044 mM; initial NaSO_4_ concentration = 50 mM; current density = 15 mA cm^−2^).

The electrolytic performance of various electrodes was assessed by the degradation of BPA using 50 mM NaSO_4_ as the supporting electrolyte. As shown in Figure 1a, after 60 min of continuous reaction, the average BPA removal percentage was 98.6%, 86.3%, and 79.3% for BDD and ACF, Pt and ACF, and BDD and Pt electrolysis systems, and 65.6% for the adsorption by BDD and ACF. Compared to the Pt and ACF and BDD and Pt system, BDD and ACF exhibited the best performance for the removal of BPA, suggesting that the synergistic effect of the BDD anode and ACF cathode was able to accelerate the generation and activation of reactive species. A pseudo-first-order model was then employed to describe the kinetic constants (K_app_) of the electrolysis systems above. As depicted in Figure 1b, the corresponding K_app_ for BDD and ACF, Pt and ACF, BDD and Pt, and BDD and ACF adsorption processes was calculated to be 0.072, 0.030, 0.025, and 0.017 min^−1^, respectively, indicating that the extraordinary performance of the BDD and ACF electrolysis system was not a simple composition of the anode oxidation and cathode activation of reactive oxygen species, as the K_app_ of BDD and ACF was folds over the other systems.

### 3.2. Process Optimization

#### 3.2.1. Effect of pH on the Degradation of BPA in the BDD and ACF System

**Figure 2:**
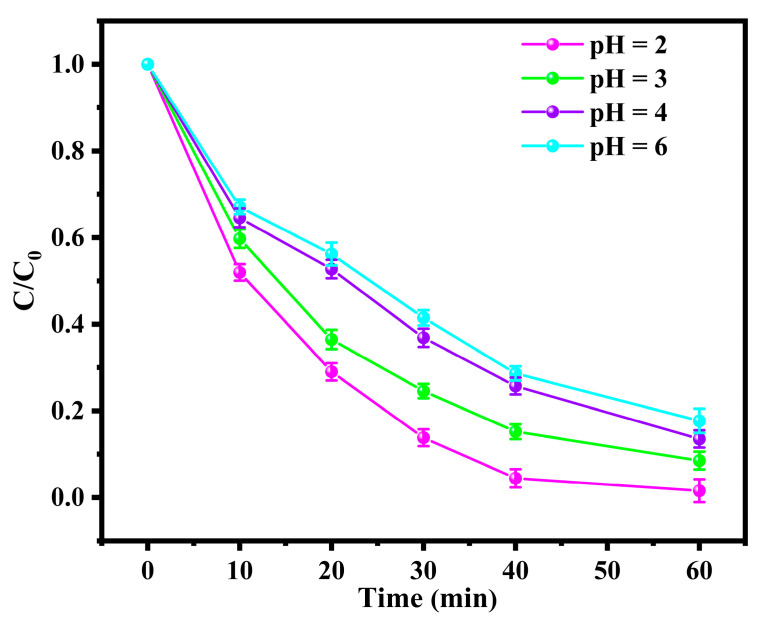
Effect of pH on the degradation of BPA in BDD and ACF system.

To estimate the influence of pH on BPA degradation within the BDD and ACF system, the pH of the solution was maintained at 2.0, 3.0, 4.0, and 6.0 (±0.2) by the addition of either H_2_SO_4_ or NaOH solution (0.1 M). As shown in Figure 2, the degradation of BPA evinced a pronounced reliance on the pH values as the removal efficiency decreased from 98.6% to 82.3% when the pH was raised from 2 to 6. The pH dependency of the reaction can be elucidated from the perspective of the two half-reactions at the anode and cathode. For the anode reaction, a higher overpotential for oxygen evolution reaction (OER) was achieved in an acidic medium, which was beneficial for the direct oxidation of pollutants [20]. For the cathode reaction, an acidic condition could not only accelerate the generation of sulfate radicals [31,32], but also enhanced the chemical adsorption between ACF and the pollutants through the formation of acidic functional groups on the surface of the ACF [33].

#### 3.2.2. Effect of Current Density on the Degradation of BPA in the BDD and ACF System

**Figure 3:**
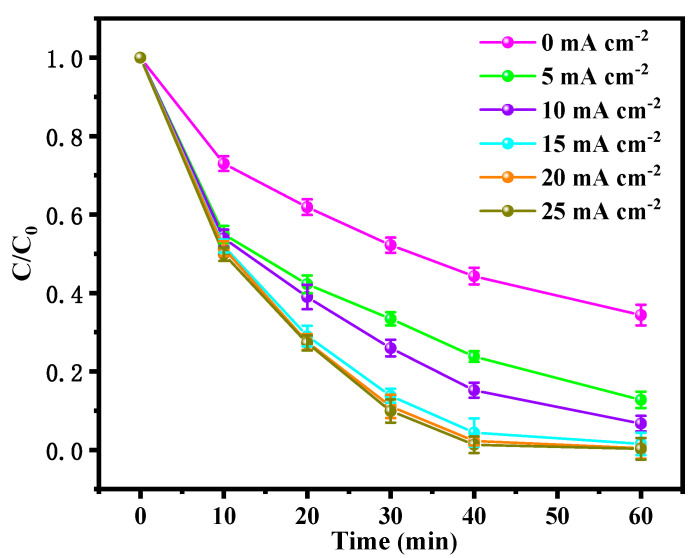
Effect of current density on the degradation of BPA in BDD and ACF system.

The current density is considered a vital parameter in an electrochemical degradation system as it represents the applied current per unit area of the electrode and provides the driving force for the redox reaction. This study investigated the effect of current density (ranging from 0 to 25 mA·cm^−2^) on the degradation of BPA within the BDD and ACF system (Figure 3). The results indicate that increasing the current density at low levels had a significant influence on the degradation of BPA as the removal efficiency showed an obvious enhancement from 65.6% to 99.6% when the applied current density increased from 0 mA·cm^−2^ to 15 mA·cm^−2^. This was due to a higher applied current density which could facilitate the efficiency of the electrochemical redox reaction, thereby leading to the enhancement of the amount of ROSs. However, when the applied current density increased to 20 mA·cm^−2^ sequentially, the enhancement of removal efficiency was negligible, which means that the increased current density did not affect the degradation of BPA significantly beyond a certain threshold value. This result was primarily due to the fact that increasing the current density requires raising the applied potential, which could lead to the occurrence of hydrogen evolution, thereby affecting the generation process of ROSs and resulting in the reduction in current efficiency [34]. Moreover, considering that the energy consumption of the reaction system was growing steadily with the increased current density, 15 mA·cm^−2^ was selected as the optimization for the BDD and ACF electrolysis system to achieve high current efficiency.

#### 3.2.3. Stability and Extensive Applicability in Environmental Remediation of the BDD and ACF System

**Figure 4:**
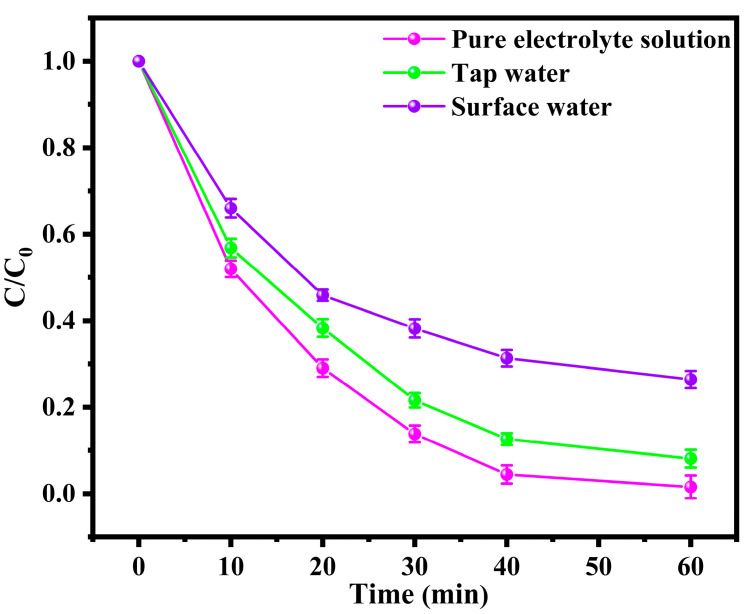
Effect of different water matrices on the degradation of BPA in BDD and ACF system.

A cycling test was conducted to evaluate the stability of the BDD and ACF system. As shown in Figure S1 and Table S1, the electrolysis system maintained a more than 90% removal of BPA after four rounds, demonstrating its stability under the applied voltage. Moreover, we also conducted the test in different water matrices to evaluate the extensive applicability in environmental remediation of the BDD and ACF system. The ramifications of matrix components present in water are multifaceted and contingent upon the unique attributes of both the matrix and the reaction system. Organic species can exert inhibitory or promotional influences, which may manifest through mechanisms such as scavenging effects, adsorption to catalysts, generation of reactive oxygen species to enhance indirect photolysis, or regeneration of the catalyst. Similarly, inorganic species may engender either inhibitory or promotional effects, potentially mediated by mechanisms such as scavenging effects, iron complexation, adsorption to the catalyst, or modulation of its effective surface area. Iron ions, for instance, may function as an additional source of catalyst, promoting reaction rates in certain contexts [35]. Therefore, it is necessary to conduct the electrolysis experiment under pure water, tap water, and surface water to investigate the mechanism of the behavior for co-existing substances. Table S2 presents a summary of the water quality parameters for the electrolyte solution, tap water, and surface water utilized in this study. The Na_2_SO_4_ electrolyte solution was produced using ultrapure water and contained exclusively Na^+^ and SO_4_^2−^ ions in the solution. The dissolved organic carbon (DOC) concentration in tap water and surface water was found to be 0.98 and 9.51 mg·L^−1^, respectively. The surface water exhibited a higher content of NH_3_-N, Cl^−^, PO_4_^3−^, and HCO_3_^−^ compared to tap water, while the other parameters were similar. The results depicted in Figure 4 indicate that the removal efficiency of bisphenol A (BPA) after 60 min of reaction was 98.6%, 92.0%, and 73.6% in pure Na_2_SO_4_ electrolyte solution, tap water, and surface water, respectively. The considerable degradation efficiency in complex water conditions underscores the broad applicability of our electrolysis system. In addition, it should be noted that the presence of organic matter and certain inorganic ions, such as chloride and bicarbonate anions, can impede the oxidation efficiency of the generated SO_4_^−^· and ·OH, thereby reducing the degradation efficiency [36].

### 3.3. Comparison of the Degradation of BPA in Divided/Undivided Cell Systems

**Figure 5:**
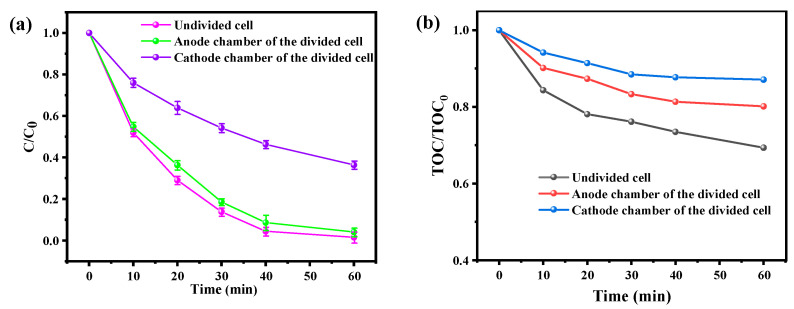
(**a**) The degradation of BPA in single/double chamber BDD and ACF systems. (**b**) The BPA mineralization performance in divided/undivided BDD and ACF systems.

To provide further insight into the individual contributions of the anode and cathodic reactions in the BDD and ACF electrolysis system, we conducted additional investigations on the degradation behavior of BPA in a divided cell configuration. The experimental set-up and electrode materials were identical to those used in the undivided cell configuration, with the exception of the incorporation of a cation exchange membrane to impede the migration of anions such as SO_4_^2−^ and S_2_O_8_^2−^ [37]. As illustrated in Figure 5a, the removal of BPA in the divided cell system was primarily attributed to anode oxidation in the anode chamber, while adsorption and activation of reactive oxygen species on ACF were responsible for the cathode chamber. Compared to the undivided cell, which achieved a BPA removal efficiency of 98.6%, the degradation efficiency was approximately 96% for the anode chamber of the divided cell, and 73.6% for the cathode chamber within 60 min. Although the degradation efficiency of the anode chamber only exhibited a slight decrease of approximately 2.6%, the average voltage of the divided cell increased significantly from 7.2 V to 25.8 V as the degradation process was operated at the same current density of 15 mA·cm^−2^, resulting in a five-fold increase in energy consumption from 0.23 kWh·m^−3^ to 1.11 kWh·m^−3^ compared to the undivided cell configuration. The poor degradation behavior of the divided cell may be attributed to the low kinetics of the anode oxidation process, which employs non-radical oxidation pathways. The assumed active species ·OH_ads_ and S_2_O_8_^2−^ were not found to exhibit strong redox capacities compared to SO_4_^2−^· and ·OH. Furthermore, the cation exchange membrane impeded the migration of anions, causing the accumulation of SO_4_^2−^ in the cathode chamber. The formation of S_2_O_8_^2−^ in the anode chamber was also impeded from reaching the surface of the ACF cathode, leading to lower charge transfer efficiency and a weak oxidizing ability for the active species in the divided cell electrolysis system. Moreover, in order to explore the BPA mineralization performance in the BDD and ACF system, total organic carbon (TOC) analysis was conducted. As shown in Figure 5b, the mineralization efficiency of BPA degradation was 31.7% (from 4.64 mg L^−1^ to 3.22 mg L^−1^) within 60 min in the undivided BDD and ACF system. However, without the synergic effect of the BDD anode and ACF cathode, the BPA mineralization performance was significantly decreased in the divided BDD and ACF system. The combined experimental results indicate that undivided BDD and ACF systems are more conducive to achieving the mineralization process of pollutants.

### 3.4. The Yield of S_2_O_8_^2−^ and H_2_O_2_ in the Electrolysis System

**Figure 6:**
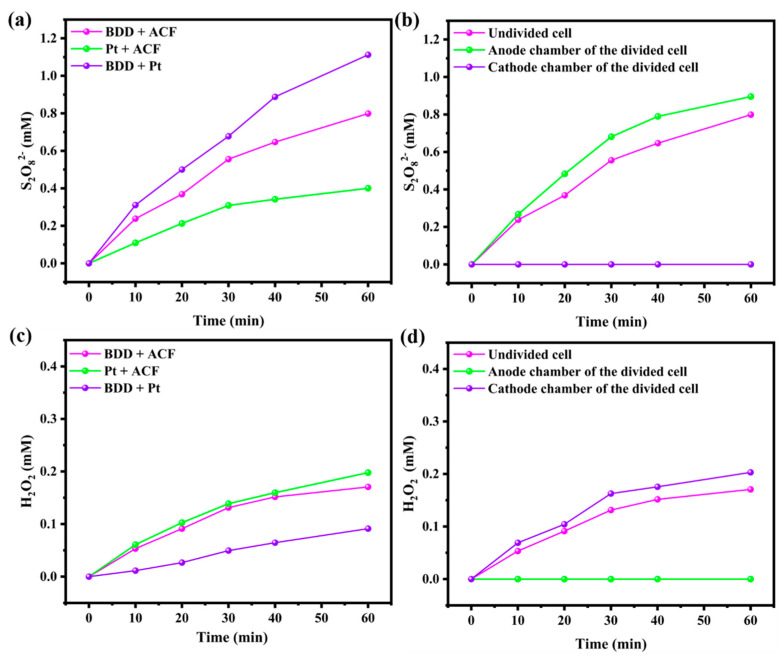
The amount of generated S_2_O_8_^2−^ (**a**) and H_2_O_2_ (**b**) in single-chamber system with different electrodes. Comparison of generated S_2_O_8_^2−^ (**c**) and H_2_O_2_ (**d**) in single/double chamber systems.

Prior research has demonstrated that BDD anodes have the capability to generate S_2_O_8_^2−^ (Equation (2)) [38], while ACF cathodes are considered a promising candidate for both H_2_O_2_ generation (Equation (3)) [39] and S_2_O_8_^2−^ activation (Equation (4)) [40]. However, the precise mechanism for the activation of these potential oxidants has yet to be fully elucidated. To address this gap, we conducted a series of measurements aimed at identifying the key active species involved in the electrolysis process to validate the findings presented in the BPA degradation section. Analysis of the results (Figure 6a) revealed that the accumulated S_2_O_8_^2−^ was calculated to be 1.11, 0.80, and 0.40 for the BDD and ACF, Pt and ACF, and BDD and Pt electrolysis systems, respectively. Comparison of the amount of S_2_O_8_^2−^ formed in the BDD and ACF and Pt and ACF systems confirmed the greater ability of BDD anodes to generate S_2_O_8_^2−^. Notably, no S_2_O_8_^2−^ was detected in the cathode chamber of the divided cell, thus confirming that the cathode did not participate in S_2_O_8_^2−^ generation. The discrepancy observed between the anode chamber of the divided cell and undivided cell indicated the diffusion of S_2_O_8_^2−^ in the solution (Figure 6b). Furthermore, more S_2_O_8_^2−^ was generated in the BDD and Pt systems compared to the BDD and ACF systems, suggesting that ACF also participated in the decomposition of S_2_O_8_^2−^. Given that ACF is recognized as an effective cathode material for H_2_O_2_ generation (Equation (3)), it could be speculated that H_2_O_2_ also played an important role in the electrolysis reaction. Indeed, as shown in Figure 6c, the amount of generated H_2_O_2_ increased from 0.09 mM to 0.17 mM when the Pt cathode was replaced with ACF, indicating the accelerated formation of H_2_O_2_ on the surface of ACF. However, when compared to the Pt and ACF system, the amount of accumulated H_2_O_2_ in the BDD and ACF system exhibited a slight decrease, and the yield in the cathode chamber of the divided cell was markedly higher than that of the undivided cell (Figure 6d). The aforementioned observations and deductions regarding the participation of ACF in the decomposition of S_2_O_8_^2−^ suggest that ACF cathodes may activate S_2_O_8_^2−^ either through direct electron transfer (Equation (4)) or via the self-generated H_2_O_2_ (Equation (5)).

2SO_4_^2−^ − 2e^−^ → S_2_O_8_^2−^ (2)

O_2_ + 2e^−^ + 2H^+^ → H_2_O_2_ (3)

S_2_O_8_^2−^ + 2e^−^ → SO_4_^−^· + SO_4_^2−^ (4)

S_2_O_8_^2−^ + H_2_O_2_ → 2SO_4_^−^· + 2·OH (5)

### 3.5. Mechanism of the Degradation of BPA in Divided/Undivided Cell Systems

**Figure 7:**
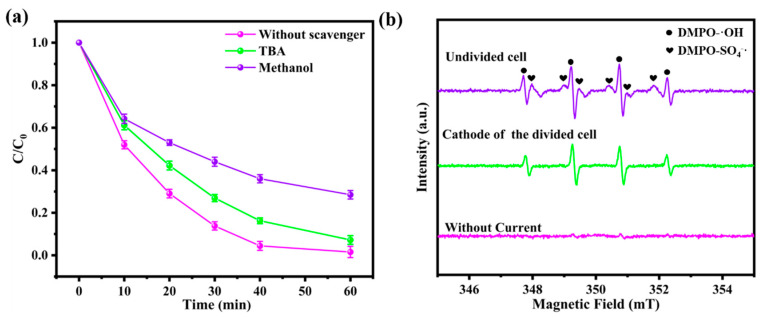
Quenching experiment (**a**) and (**b**) EPR spectrum of the BDD and ACF system.

The self-circulation mechanism of sulfate and persulfate ions during the electrolysis process was investigated through a series of control experiments. Figure S2 demonstrates a significant decline in the degradation kinetics of BPA when the NaSO_4_ electrolyte was replaced with NaNO_3_ while maintaining a constant initial conductivity of 5.9 mS/cm^2^. This finding provides direct evidence that sulfate ions acted as the initiator of the self-circulation process. Quenching experiments and electron paramagnetic resonance (EPR) spectroscopy were subsequently conducted to investigate the reactive radicals during the electrolysis process. To distinguish the contributions of ·OH and SO_4_^−^· to BPA degradation, methanol and TBA were used as radical scavengers. TBA is highly reactive with ·OH only (1 × 10^9^ M^−1^·s^−1^ for ·OH and 1 × 10^5^ M^−1^·s^−1^ for SO_4_^−^·) [41], whereas methanol is highly reactive with both ·OH and SO_4_^−^· (8–10 × 10^8^ M^−1^·s^−1^ for ·OH and 0.1–1.3 × 10^7^ M^−1^·s^−1^ for SO_4_^−^·) [42]. Therefore, methanol and TBA can compete with the BPA for free radicals in an aqueous solution. The inhibition of BPA removal by both methanol and TBA in the BDD and ACF electrolysis system (Figure 7a) suggested that SO_4_^−^· radicals played an essential role in the degradation process and that ·OH was likely generated from the reaction of Equation (5). Moreover, the undivided system exhibited a significant signal of DMPO-SO_4_^−^· at the cathode compared to the divided cell (Figure 7b), confirming the direct occurrence of the reactions in Equations (3)–(5). Importantly, the removal of BPA maintained a decent rate even with the addition of excessive amounts of methanol or TBA, indicating that the protonated ACF surface promoted chemical adsorption for the negative charges of BPA, reducing the mass transfer distance between the generated radicals from the ACF cathode and the target pollutants, as consistent with the effect of pH in BPA degradation.

**Figure 8:**
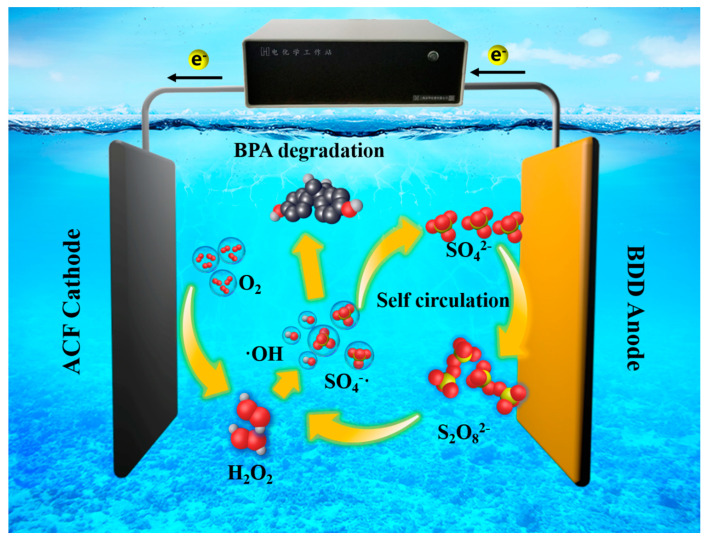
Mechanism of the BDD and ACF electrolysis system.

Based on the experimental findings and discussions above, a comprehensive understanding of the reaction pathways involved in the degradation of BPA is established, as shown Figure 8. In the presence of an applied voltage, the surface of the boron-doped diamond (BDD) anode undergoes an oxidation process, resulting in the formulation of S_2_O_8_^2−^ through the oxidation of sulfate. Subsequently, S_2_O_8_^2−^ is transmitted to the activated carbon fiber (ACF) cathode, where it is decomposed into SO_4_^−^· radicals and serves as a facilitator for the degradation of adsorbed target pollutants. This process involves a self-circulation mechanism of sulfate and persulfate, which is contributed to by the synergistic effect between the BDD anode and ACF cathode, utilizing SO_4_^−^· as an intermediate species.

### 3.6. Proposed Pathways of BPA Degradation

**Figure 9:**
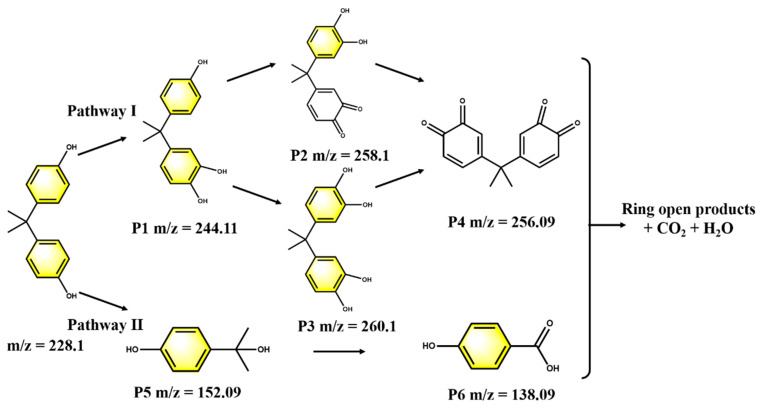
Proposed pathways of BPA degradation.

Liquid chromatography–mass spectrometry (LC-MS) analysis was employed to acknowledge the intermediates of BPA in the BDD and ACF electrolysis system (Figure 9 and Figure S3). Initially, the activation of S_2_O_8_^2−^ engendered sulfate radicals, which subsequently underwent attack on BPA and seized an electron from the aromatic ring, leading to the consequent production of phenolic radicals [43]. After that, the nucleophilic attack of phenolic radicals by ·OH could occur under acidic conditions (Pathway I), resulting in the mono (P1) or multihydroxylation (P3) of the aromatic rings. The hydroxylated BPA then underwent dehydration, forming quinone compounds (P2 and P4) [44]. On the other hand, the C-C bond, which is situated between the isopropyl and benzene rings in a para-position, exhibits susceptibility to sulfate radicals due to its elevated frontier electron density (Pathway II) [45]. As a result, BPA radicals were decomposed and oxidized to form hydroxybenzoic acid (P5-P6). Ultimately, the aromatic compounds underwent a transformation, resulting in the formation of ring-open products that could subsequently undergo oxidative cleavage, leading to their eventual mineralization.

### 3.7. Toxicity Assessment

**Figure 10:**
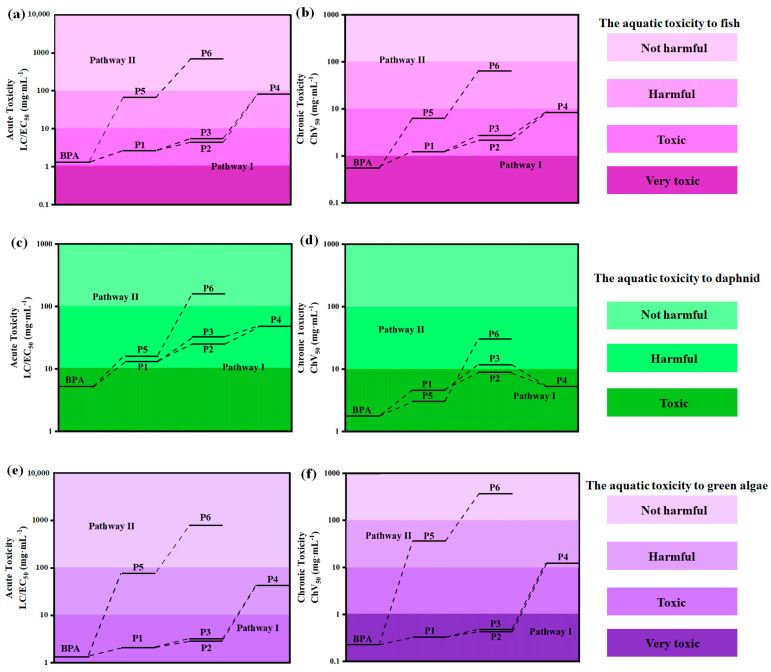
Toxicity of BPA and its degradation intermediates. The acute and chronic toxicity to fish (**a**,**b**); The acute and chronic toxicity to daphnid (**c**,**d**); The acute and chronic toxicity to green algae (**e**,**f**).

The acute and chronic toxicity of BPA and its generated intermediates during the degradation process to three selected aquatic organisms (fish, daphnid, and green algae) were predicted by the ecological structure activity relationship (ECOSAR) program. Based on the United Nations Globally Harmonized System, the classification of acute and chronic toxicities can be defined as very toxic, toxic, harmful, and not harmful (Table S3) [46]. According to Figure 10 and Table S4, BPA was categorized as “very toxic” to “toxic” (LC_50_/EC_50_/ChV < 10 mg L^−1^) to aquatic organisms in terms of both acute and chronic toxicity, and the intermediates generated during BPA degradation exhibited lower ECOSAR-predicted toxicity (P1–P7), indicating that the ecological threat was decreased after the electrolysis process. Moreover, compared to Pathway I (P1–P4), the intermediates detected in Pathway II (P5 and P6) were less toxic, demonstrating that polycyclic aromatic hydrocarbons were more toxic than monocyclic aromatic hydrocarbons during the BPA removal process. This observation revealed that the participation of SO_4_^−^· during BPA degradation could decrease the ecotoxicity effectively, thus emphasizing the unique advantage of the self-circulation of sulfate and persulfate ions.

### 3.8. Cost Evaluation

To assess the economic viability of wastewater treatment, a comprehensive cost analysis of the BDD and ACF system was conducted. The process was based on the optimum conditions previously obtained. The number of annual working days (D) and the number of working hours per day (t_w_) are assumed to be 300 d and 12 h, respectively. The reaction time is assumed to be 30 min. An additional time of 30 min is assumed for filling, emptying, and preparing the reactor for each batch process, so the total batch time (t_b_) is 1 h. The reactor capacity (V_c_) is estimated according to Equation (6) [47].

(6) $$V_{c}=\frac{V_{t}t_{b}}{Dt_{w}}$$

where V_t_ is the total volume of annually treated effluent. Assuming V_t_ is 10,000 m^3^, the calculated value for V_c_ is 2.8 m^3^. The total cost of the treatment process (TC) is determined by taking into account both the amortization cost of the investment (AC) and the operating cost (OC). The AC can be estimated by Equations (7) and (8) [48].

(7) $$A=\frac{C_{m0}{\left(1+i\right)}^{L}i}{{\left(1+i\right)}^{L}-1}$$

(8) $$AC=\frac{(C_{m0}+LA)V_{c}}{LV_{t}}$$

where C_m0_ is the cost of the BDD and ACF system’s construction and permanent facilities, which is estimated to be USD 1000. L is the lifetime of the BDD and ACF system, assumed to be 10 years. A is the annual investment cost of C_m0_; i is the annual interest rate (assumed 5%). The AC of the treatment is calculated to be 0.064 USD/m^3^.

The OC encompasses expenditures related to BDD and ACF electrodes, chemicals, energy consumption, and maintenance. The cost of replacing the BDD and ACF electrodes every year (C_e_) is 0.8 USD m^−3^. C_ch_ was estimated to be 0.30 USD m^−3^ including sodium persulfate and pH adjustment if required. The energy consumed (EC) for the operation of the DC power supply, mixer, and aeration was measured in USD m^−3^ according to Equation (9).

(9) $$EC=\frac{EDt_{w}P_{E}}{V_{t}}$$

where E denotes the total of consumed power, assumed to be 10 kW, P_E_ is the electric energy unit price (0.08 USD kW^−1^h^−1^). Additionally, the maintenance cost was presumed to be 2% of the activated carbon (AC) cost. Therefore, the comprehensive operating cost can be appraised according to Equation (10).

(10) $$OC=C_{e}+C_{ch}+EC+0.02AC$$

The calculated EC amounts to 0.29 USD m^−3^, resulting in an overall OC of 1.39 USD m^−3^. Consequently, the total cost, inclusive of amortization and operational expenses, was ascertained to be 1.454 USD m^−3^.

The economic and operational advantages observed in the treatment process firmly establish the BDD and ACF system as an efficient and cost-effective supplementary method for certain wastewater chemical treatment approaches. Its potential extends to replacing traditional electrolysis systems in pretreating influent wastewater, presenting an economically viable and efficient solution for enhancing the pretreatment process.

## 4. Conclusions

In summary, the current work investigates an electrolysis system that employs a BDD anode and an ACF cathode for the electrochemical degradation of endocrine disruptors. The crucial intermediates, S_2_O_8_^2−^ and H_2_O_2_, originated from the electrochemical redox reaction upon the BDD anode and ACF cathode, respectively, giving rise to the generation of ·OH and SO_4_^−^·, which served as the principal reactive oxygen species (ROSs) involved in the degradation process of BPA. Of novelty to our work is the self-circulation of sulfate and persulfate ions in the electrolysis system achieved by the synergistic effect between the BDD anode and ACF cathode, making it completely free from the addition of chemical reagents. The involvement of electrogenerated ·OH and SO_4_^−^· in the circulation are substantiated by quantitatively measured S_2_O_8_^2−^, quenching experiments, and the EPR spectrum. Meanwhile, the superiority of this self-circulated BDD and ACF system was proved by the significantly enhanced BPA degradation efficiency over the divided cell and the control system containing inert Pt electrodes. On the basis of LC-MS and ECORSAR analysis, the BDD and ACF system demonstrated significant efficacy in reducing the potential ecotoxicity when compared to the initial state of BPA. Our work denotes the efficacious execution of the “catalysis in lieu of supplementary chemical agents” principle in the domain of sustainable chemistry, which offers a new horizon for the design and optimization of electrolysis systems.

## Data Availability

Data will be made available on request.

## Supplementary Materials

The following supporting information can be downloaded at: https://www.mdpi.com/article/10.3390/toxics12020156/s1, Figure S1: Multi-pollutants removal experiment in 60 min in BDD and ACF system. Figure S2: Cycling test for the degradation of BPA in BDD and ACF system. Figure S3: The degradation of BPA in BDD and ACF system with NaSO_4_/NaNO_3_ as the electrolyte with a same conductivity of 5.9 mS/cm^2^. Figure S3: BPA degradation intermediates detected in the BDD and ACF system. Table S1: The energy consumption of different electro-catalytic system. Table S2: Water quality parameters of the pure electrolyte solution, tap water and surface water. Table S3: Toxicity classification according to the Globally Harmonized System of Classification and Labelling of Chemicals (GHS). Table S4: Predicted acute and chronic toxicity of SMX and its products.
