# Supplementary material for: Sustainable Electrochemical Activation of Self-Generated Persulfate for the Degradation of Endocrine Disruptors: Kinetics, Performances, and Mechanisms
Source: Toxics. 2024 Feb 17;12(2):156. doi: 10.3390/toxics12020156 (PMC10893448; doi:10.3390/toxics12020156)
Supplement: Supplementary file 1 [file toxics-12-00156-s001.zip › toxics-2849275-supplementary.pdf]

Sustainable Electrochemical Activation of Self-Generated Persulfate for the Degradation of Endocrine Disruptors: Kinetics, Performances, and Mechanisms

Xiaofeng Tang [1], Zhiquan Jin [1], Rui Zou [1], Yi Zhu [1], Xia Yao [1], Mengxuan Li [1], Shuang Song [1], Shuangliu Liu [2,], Tao Zeng [1,3,*]*

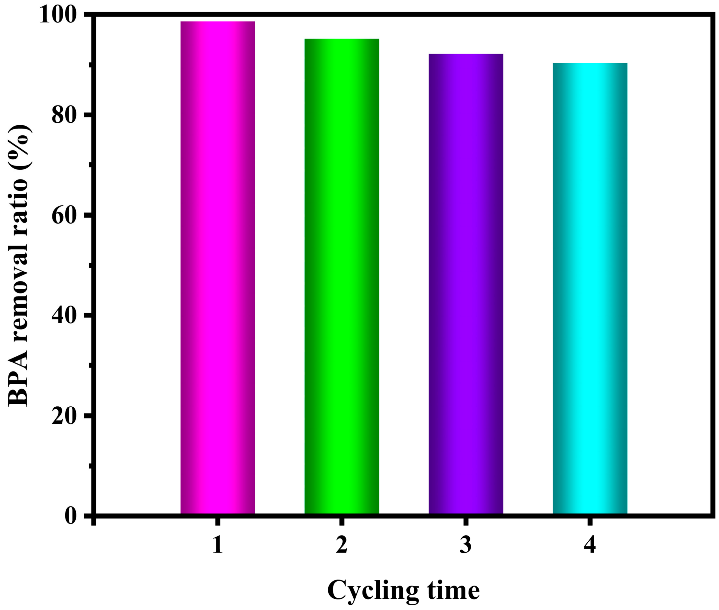

Figure S1. Cycling test for the degradation of BPA in BDD and ACF system.

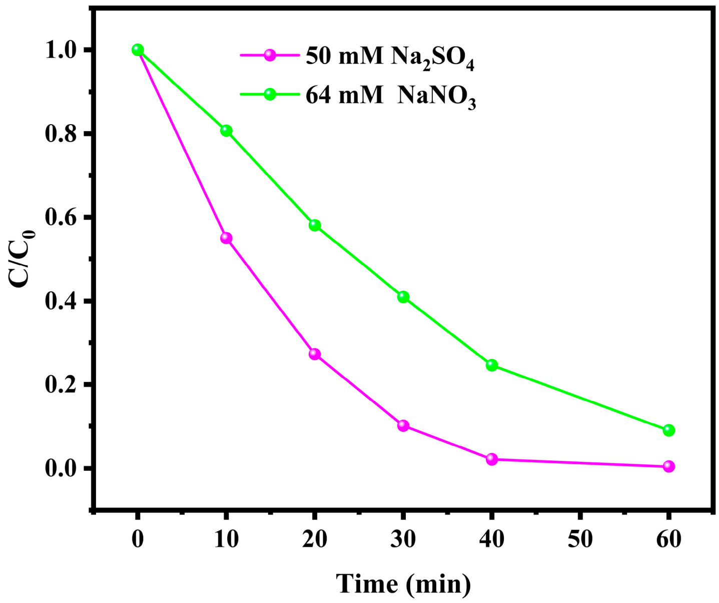

Figure S2. The degradation of BPA in BDD and ACF system with $NaSO_4/NaNO_3$ as the electrolyte with a same conductivity of 5.9 mS/cm^2.

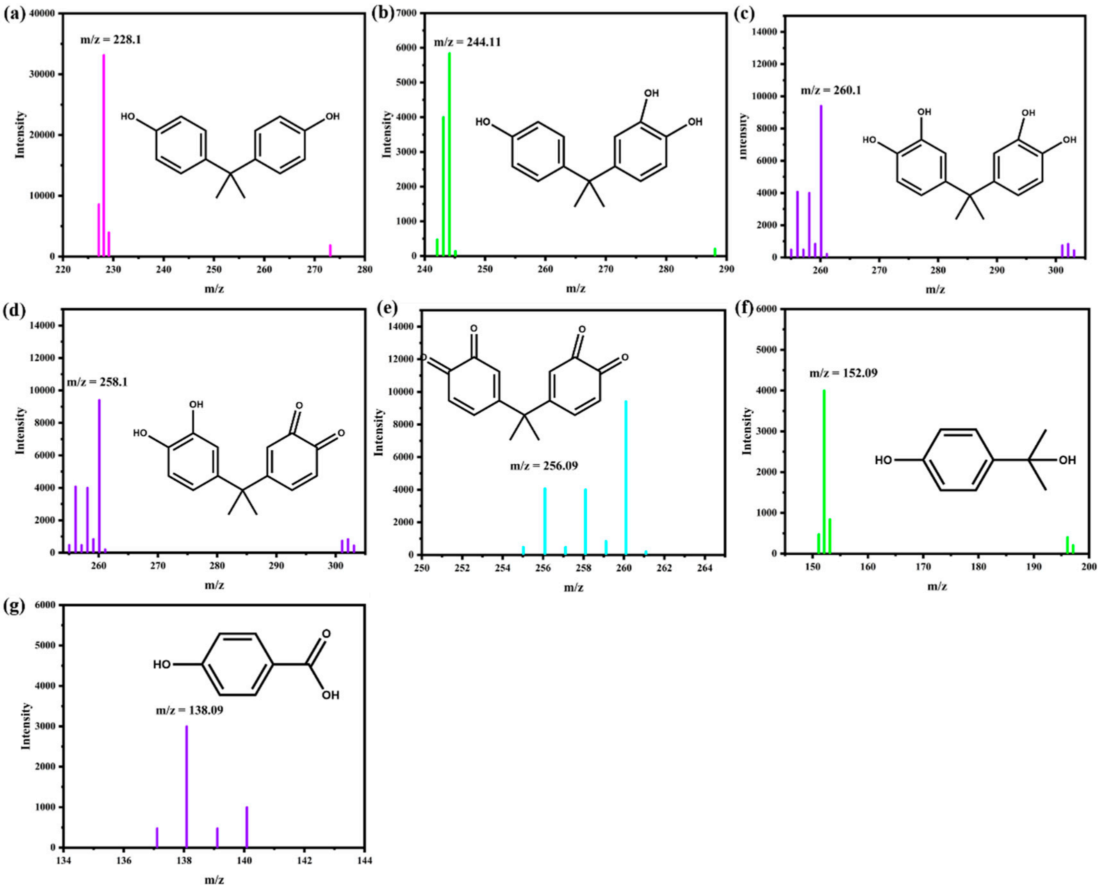

Figure S3. BPA degradation intermediates detected in the BDD and ACF system.

Table S1. The energy consumption of different electro-catalytic system.

Reaction system	Current density ($mA\cdot cm^{-2}$)	Average voltage (V)	The energy consumption ($kWh\cdot m^{-3}$)
	5	4.3	0.094
	10	5.8	0.19
Undivided cell	15	7.2	0.23
	20	8.5	0.28
	25	9.8	0.38
Anode chamber of the divided cell	15	25.8	1.11

Table S2. Water quality parameters of the pure electrolyte solution, tap water and surface water.

Constituent	Pure electrolyte solution	Tap water	Surface water
DOC (mg/L)	0	0.98	9.51
CO_3^{2-} (mg/L)	-	0.93	2.23
HCO_3^- (mg/L)	-	58	170
Cl^- (mg/L)	-	5.3	13.5
SO_4^{2-} (mg/L)	4800	5098	5226
PO_4^{3-} (mg/L)	-	1.83	4.35
NO_3^- (mg/L)	-	1.23	1.04
Ammonia (mg-N/L)	-	-	3.56

Table S3. Toxicity classification according to the Globally Harmonized System of Classification and Labelling of Chemicals (GHS).

Toxicity range (mg L^{-1})	Class
$LC_{50}/EC_{50}/ChV \leq 1$	Very toxic
$1 < LC_{50}/EC_{50}/ChV \leq 10$	Toxic
$10 < LC_{50}/EC_{50}/ChV \leq 100$	Harmful
$LC_{50}/EC_{50}/ChV > 100$	Not harmful

Table S4. Predicted acute and chronic toxicity of SMX and its products

Compound	Acute toxicity (mg L^{-1})			Chronic toxicity (ChV) (mg L^{-1})		
	Fish (LC_{50})	Daphnid (LC_{50})	Algae (EC_{50})	Fish	Daphnid	Algae
BPA	1.284	5.237	1.331	0.550	1.773	0.227
P1	2.655	13.105	2.070	1.224	4.568	0.329
P2	4.435	24.998	2.837	2.152	8.892	0.429
P3	5.466	32.653	3.205	2.712	11.718	0.474
P4	81.312	48.173	42.773	8.356	5.287	12.313
P5	67.265	15.969	77.359	6.280	3.041	36.588
P6	692.680	159.249	776.856	63.989	30.325	367.767

Not harmful   Harmful   Toxic   Very toxic
